# Functional modulation of LHCSR1 protein from *Physcomitrella patens* by zeaxanthin binding and low pH

**DOI:** 10.1038/s41598-017-11101-7

**Published:** 2017-09-11

**Authors:** Alberta Pinnola, Matteo Ballottari, Ilaria Bargigia, Marcelo Alcocer, Cosimo D’Andrea, Giulio Cerullo, Roberto Bassi

**Affiliations:** 1 Dipartimento di Biotecnologie, Università di Verona, Strada Le Grazie 15, I-37134 Verona, Italy; 2 Center for Nano Science and Technology @PoliMi, Istituto Italiano di Tecnologia, via Pascoli 70/3, 20133 Milano, Italy; 3 IFN-CNR, Department of Physics, Politecnico di Milano, P.za L. da Vinci 32, 20133 Milano, Italy; 4 Consiglio Nazionale delle Ricerche (CNR), Istituto per la Protezione delle Piante (IPP), Via Madonna del Piano 10, 50019 Sesto Fiorentino Firenze, Italy

## Abstract

Light harvesting for oxygenic photosynthesis is regulated to prevent the formation of harmful photoproducts by activation of photoprotective mechanisms safely dissipating the energy absorbed in excess. Lumen acidification is the trigger for the formation of quenching states in pigment binding complexes. With the aim to uncover the photoprotective functional states responsible for excess energy dissipation in green algae and mosses, we compared the fluorescence dynamic properties of the light-harvesting complex stress-related (LHCSR1) protein, which is essential for fast and reversible regulation of light use efficiency in lower plants, as compared to the major LHCII antenna protein, which mainly fulfills light harvesting function. Both LHCII and LHCSR1 had a chlorophyll fluorescence yield and lifetime strongly dependent on detergent concentration but the transition from long- to short-living states was far more complete and fast in the latter. Low pH and zeaxanthin binding enhanced the relative amplitude of quenched states in LHCSR1, which were characterized by the presence of 80 ps fluorescence decay components with a red-shifted emission spectrum. We suggest that energy dissipation occurs in the chloroplast by the activation of 80 ps quenching sites in LHCSR1 which spill over excitons from the photosystem II antenna system.

## Introduction

Eukaryotic photosynthetic organisms harvest photons using chlorophyll- and carotenoid-binding proteins called light-harvesting complexes (LHCs)^1–4^. The gene products of LHC’s multigene family share a basic structural organization including three transmembrane α-helices and short amphipathic helices at the lumenal surface^5–9^, with chlorophylls (Chls) and carotenoid (Car) binding sites partially conserved among the different family members. A Chl a cluster has been reported at the core of the holoproteins, close to the inner helices A and B, while Chl b binding sites are localized more peripherally, connected to the helix C and the C-terminal domain^5–9^. In addition, the affinity of individual binding sites for Chl a vs Chl b ligands is variable among LHC complexes^5, 6, 10^. Two conserved carotenoid binding sites, called L1 and L2, are located parallel to the A and B helices for all LHC proteins, while two additional sites per monomer, called N1 and V1, were reported in the trimeric LHCII^5^. Other LHC members lack N1, V1 or both^6–9, 11, 12^. LHC subunits can reversibly switch between conformations with different Chl fluorescence lifetimes, which, depending on trapping time of the reaction centers, correspond to light-harvesting or energy-dissipation modes of their function, the latter being crucial for preventing over-excitation and the consequent oxidative damage of the photosynthetic apparatus^13–18^. Conformational switch is controlled by thylakoid lumenal pH: when photosynthetic electron transport is saturated, and ATPase activity is inhibited by the lack of its substrate, ADP, protons over-accumulate in the lumen, triggering thermal dissipation of the excess absorbed energy by a mechanism called non-photochemical quenching (NPQ). The sensors of lumen acidification are the PSBS protein in higher plants and the LHCSR proteins in unicellular algae, which both harbor specific acidic residues, undergoing protonation, essential for activity^16, 17, 19–23^. LHCSR proteins and PSBS coexist in lower plants, and both contribute to NPQ activity^24^. Transient accumulation of PSBS has been recently reported in *Chlamydomonas reinhardtii* (*C*. *reinhardtii*), upon exposure to high light^25–27^. The major difference between LHCSR and PSBS proteins consists in the fact that the former binds both Chls and xanthophylls while the latter does not. Thus, the activation mechanisms of NPQ by the two proteins need to be distinct: the establishment of intramolecular pigment-pigment interactions leading to energy dissipation can only occur in LHCSR proteins^16, 17, 20, 28^. PSBS, instead, although active in pH detection, requires interacting pigment-binding proteins as sites(s) for quenching reactions. Consistently, a low pH-dependent switch to a dissipative state has been demonstrated in LHCSR proteins from both *C*. *reinhardtii* (C.r.LHCSR1 and C.r.LHCSR3)^29^ and *Physcomitrella patens* (P.p.LHCSR1)^16, 17, 20, 28^.

Besides direct protonation of PSBS/LHCSR proteins, additional regulation of NPQ activity is provided by the xanthophyll cycle, consisting in reversible epoxidation of zeaxanthin (Zea) in plants and algae, or diatoxanthin in diatoms^30, 31^. Low lumenal pH activates the enzyme Violaxanthin de-epoxidase (VDE) which converts violaxanthin (Vio) into Zea^32^, up-regulating both LHCSR- and PSBS-dependent NPQ activities^33, 34^. Zea accumulation was reported to increase photoprotection through multiple mechanisms, including scavenging of Reactive Oxygen Species and quenching of chlorophylls singlet and triplet excited states^11, 33, 35–42^. Zea has been reported to substitute for Vio at the external V1 site in LHCII trimers and at the internal L2 site in monomeric LHC complexes^5, 6, 11, 36, 38, 42–45^, the latter location being effective in switching LHC monomers to a short lifetime conformation^13, 36, 38, 42, 46, 47^. As for LHCSR proteins, the effect of Zea on quenching activity is species-specific: in *C*. *reinhardtii*, Zea synthesis does not significantly modulate LHCSR quenching properties^16, 28^ and indeed the NPQ induction in *C*. *reinhardtii* is Zea-independent^16, 48^. Differently, in *P*. *patens*, NPQ activation by both LHCSR and PSBS proteins was shown to be strongly dependent on Zea^33^. Recently, LHCSR1 from *P*. *patens* has been reported to host a Zea-dependent quenching mechanism triggered at low pH involving energy transfer from chlorophyll to the carotenoid S1 state^29^. In addition a Zea-independent quenching mechanisms has been also reported for both P.p.LHCSR1 and C.r.LHCSR3, involving formation of carotenoid radical cations^16, 29^. In this work, we have studied the optical properties of P.p.LHCSR1 produced by overexpression in tobacco in either its Vio- or Zea-binding forms and purified either directly from dark-adapted leaves or upon incubation at low pH which activates Vio de-epoxidation. By using steady-state and time-resolved spectroscopic methods, we show that acidification of the medium and protein-protein interactions induce fluorescence quenching in P.p.LHCSR1 to a far higher extent compared to the homologous trimeric LHCII antenna protein, leading to the formation of a red-shifted spectral component with lifetime below 100 ps, which can act as a suitable quencher for LHC complexes. The binding of Zea further decreased fluorescence lifetime of this pigment-protein complex and enhanced the sensitivity of quenching to low pH.

## Results

### Biochemical characterization of LHCSR1 isolated proteins

LHCSR1 fraction eluted from affinity column was pure, as judged from the absence of contaminant proteins detectable in Coomassie stained SDS-PAGE gel^49^. Pigment binding properties of LHCSR1 purified from control (ctrl) and de-epoxidated (dep) thylakoids were investigated by absorption spectroscopy of acetone pigment extracts and HPLC^50^ (Table 1). Both LHCSR1-crtl and LHCSR1-dep showed a very high Chl a/b ratio (>60), implying that Chl b is largely sub-stoichiometric compared to the LHCSR1 apoprotein. Based on 8 chlorophylls per apoprotein^16, 28, 49^, only ~12–15% of the LHCSR1-ctrl and LHCSR1-dep holoproteins respectively bind one Chl b molecule, while the remaining proteins bind Chl a only. Both LHCSR1-ctrl and LHCSR1-dep were characterized by a Chl/Car ratio of 2.1–2.05, i.e. ~3.8 carotenoids per apoprotein. LHCSR1-ctrl bound close to two (1.95) luteins (Lute) per polypeptide as well as two (1.85) Vio and traces of antheraxanthin. LHCSR1-dep showed essentially the same Chl/Car ratio while both Lute and Vio complement decreased to 1.53 and 0.79, respectively, being substituted for by 1.36 Zea and 0.22 antheraxanthin (Table 1) leading to a de-epoxidation index of 0.62. Thus, Zea substituted for both Vio and, to a lower extent, for Lute.

**Table 1:** Pigments analysis of recombinant LHCSR1 proteins isolated from tobacco plants.

	tot Chls	Chla/Chlb	tot Cars	Chls/Cars	Chla	Chlb	neo	viola	anthera	lute	zea	b car
LHCSR1 CTRL	8.00	79.82	3.80	2.10	7.88	0.12	n.d	1.77	0.08	1.95	n.d	n.d
*s*.*d*.		*51*.*18*	*0.10*	*0*.*05*	*0*.*08*	*0*.*08*		*0*.*06*	*0*.*01*	*0*.*03*		
LHCSR1 DEP	8.00	60.85	3.89	2.05	7.85	0.15	n.d	0.79	0.22	1.53	1.36	n.d
*s*.*d*.		*29*.*43*	*0*.*24*	*0*.*13*	*0*.*07*	*0*.*07*		*0*.*23*	*0*.*18*	*0*.*11*	*0*.*28*	

LHCSR1 CTRL: LHCSR1 protein isolated from tobacco thylakoids purified form dark adapted plants. LHCSR1 DEP: LHCSR1 protein isolated from tobacco thylakoids de-epoxidized *in vitro* in order to induce zeaxanthin accumulation.

**Figure 1:**
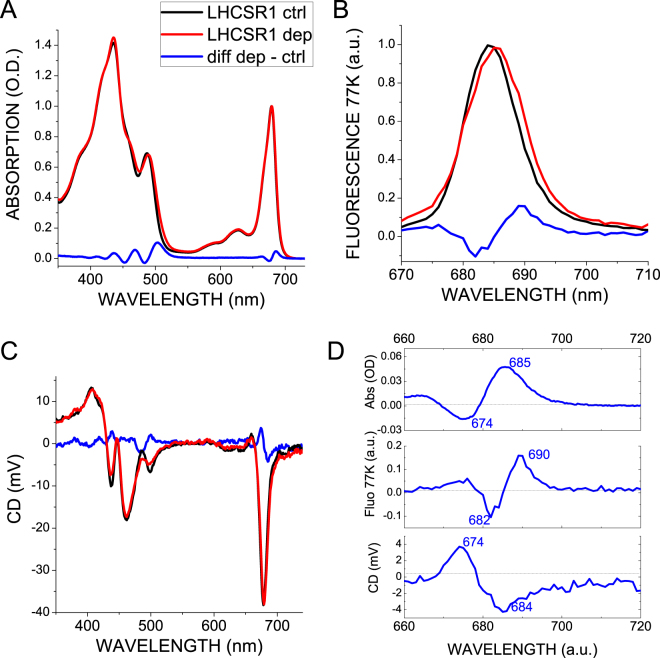
Spectroscopic properties of LHCSR1-ctrl and LHCSR1-dep. Absorption (**A**), 77 K fluorescence emission (**B**) and circular dichroism spectra are reported for LHCSR1-ctrl (black) and LHCSR1-dep (red). Difference spectra LHCSR1-dep *minus* LHCSR1-ctrl are reported in each panel and summarized in Panel D.

Absorption spectra of LHCSR1-ctrl and LHCSR1-dep are reported in Fig. 1: the presence of Zea in LHCSR1-dep caused an increased amplitude of the 500 nm shoulder in the Soret band, due to the red-shifted absorption of Zea vs Vio and Lute, as previously reported for other members of the LHC family^11, 51, 52^. Zea binding also caused a red shift of chlorophyll absorption in the Qy region, indicating the formation of chlorophyll transitions with lower energy (Fig. 1). Consistently, the fluorescence emission spectrum at 77 K of LHCSR1-dep was further red shifted compared to LHCSR1-ctrl (Fig. 1B). The formation of Zea-dependent low-energy transitions was previously reported to be related to an enhanced excitonic interaction between Chl a molecules^36, 38^. Consistently, the amplitude of the Qy signal in circular dichroism spectra was enhanced in presence of Zea (Fig. 1C) showing a negative/positive signal at 674/685 nm (Fig. 1D), suggesting a stronger interaction between pigments^16, 38, 53^.

**Figure 2:**
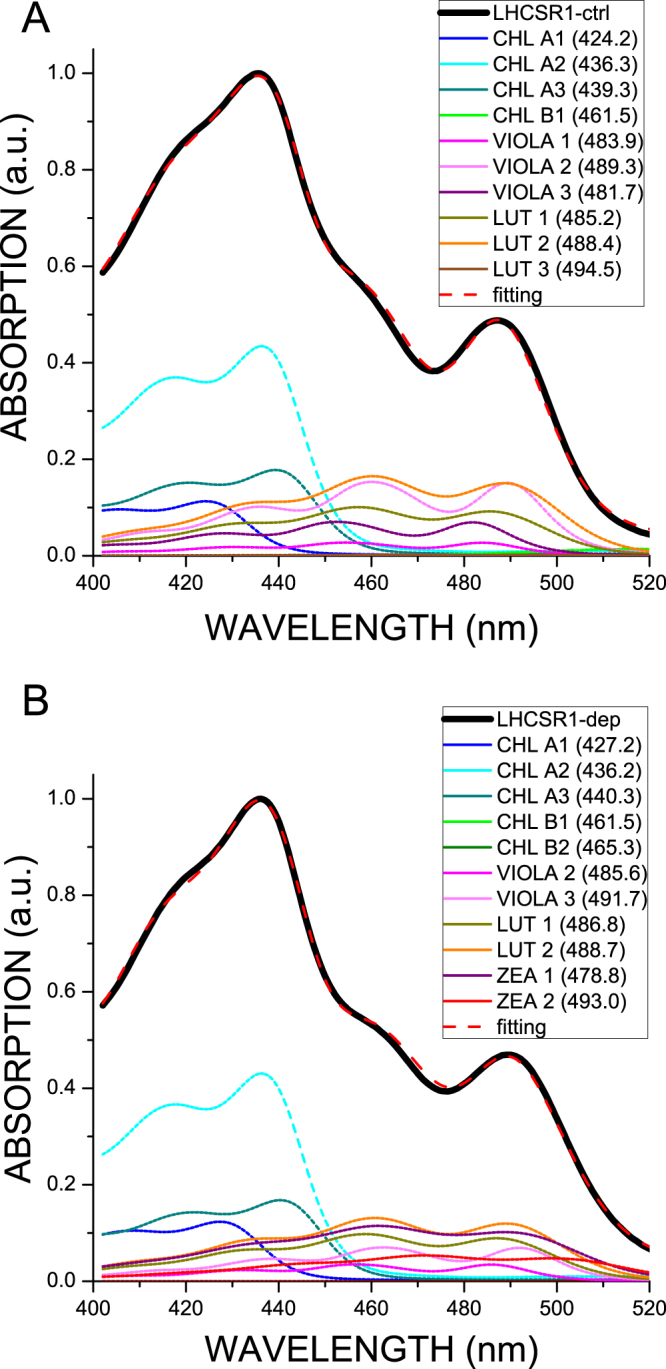
Spectral deconvolution of Soret absorption spectra of LHCSR1 control and after de-epoxidation *in vitro*.

Carotenoid absorption is strongly affected by binding to different sites within LHC proteins^45, 54^. In order to gain information on the multiple occupancy of binding sites by xanthophylls, in particular by Zea, within LHCSR1, the absorption spectra in the Soret region of LHCSR1-ctrl and LHCSR1-dep were fitted with Chls and Cars absorption forms as previously described^16, 54^. Indeed, the transition energies of xanthophylls ligands are strongly affected by the refraction index of the medium^54^ and ligation to different protein sites where xanthophylls are either buried in the protein structure (sites L2, L2) or more exposed to the solvent (sites V1, N1) reflects into differential shifting with respect to the reference value in 80% acetone. In the case of LHCSR1-ctrl, best fitting was obtained with three Chl a, one Chl b, three Lute and three Vio spectral forms with a Chl a/b ratio of 28.4, a Chl/Car ratio of 2.5 and a Vio/Lute ratio of 0.88 (Fig. 2). This result closely fits the data from HPLC pigment analysis (Table 1). The different carotenoid spectral forms could be divided into three groups according to the amplitude of their shift in the 0–0 absorption wavelength with respect to the value in organic solvent: shifts were of 10.9 nm, 16.3–17 nm and 7.7–8.7 nm, respectively, that can be associated to spectral tuning induced respectively by binding to L1, L2 and N1/V1-like carotenoid sites as for previous analysis in members of the LHC family^16, 54^. According to this model, all carotenoid binding sites are occupied by both Lute and Vio with site occupation ratios of 87% *vs*13% (L1); 1% vs 99% (L2); 40% - *vs* 60% (N1-V1) respectively (Table 2). Lute and Vio are thus the major ligands of L1 and L2 sites respectively, consistent with previous data reported for the monomeric CP29 protein^6^. Lute and Vio were as well bound to other LHCSR1 sites yielding a lower value of red shift similar to what was previously reported for N1 or V1 sites. In the case of LHCSR1-dep best fitting was obtained by using, again, three Chl a, two Chl b and six carotenoid spectral forms: two for Lute, two for Vio and two for Zea. The shifts applied to carotenoid spectral forms were similar to those used for LHCSR1-ctrl, allowing for identification of L1 (with a 11.2–12.6 nm shift), L2 (15.5–18.7 nm) and N1/V1-like (9.3–5.8 nm) sites with Zea spectral forms having shift values consistent with L2 and N1/V1-like sites, but not L1 site. Thus, the L1 site in LHCSR1-dep appeared to retain the composition as in the control protein: Lute (80%) and Vio (20%). The 15.5–18.7 nm shifted spectral forms corresponding to the L2 site, consisted of Vio (53%) and Zea (47%), while the least-shifted forms consistent with N1/V1-like sites were represented by Lute (44%) and Zea (56%) (Table 2). These results indicate that the de-epoxidation of Vio in the more peripheral carotenoid binding sites (N1/V1-like sites) was almost complete, while only roughly half of the Vio in L2 underwent conversion into Zea. Interestingly, Vio in L1 appeared not to be available for de-epoxidation. In agreement with HPLC pigment analysis, de-epoxidation induced a loss of Lute from N1/V1 sites of LHCSR1-dep.

**Table 2:** Results of Soret absorption deconvolution.

Cars Binding Sites		LHCSR1-ctrl		LHCSR1-dep		
		LUT	VIOLA	LUT	VIOLA	ZEA
L1	*shift*	10.9	10.9	11.2	12.6	n.d.
	*%*	87%	13%	80%	20%	n.d.
L2	*shift*	17.0	16.3	n.d.	18.7	15.5
	*%t*	1%	99%	n.d.	53%	47%
N1/V1	*shift*	7.7	8.7	9.3	n.d.	5.8
	*%*	60%	40%	44%	n.d.	56%

LHCSR1-ctrl and LHCSR1-dep absorption spectra were fitted in the Soret region with chlorophylls and carotenoid spectral forms. Shift values obtained for the different carotenoid spectral forms are reported and clustered according to similar values. The resulting distribution of carotenoid binding site occupancy is reported in %.

### Fluorescence properties of LHCSR1

**Figure 3:**
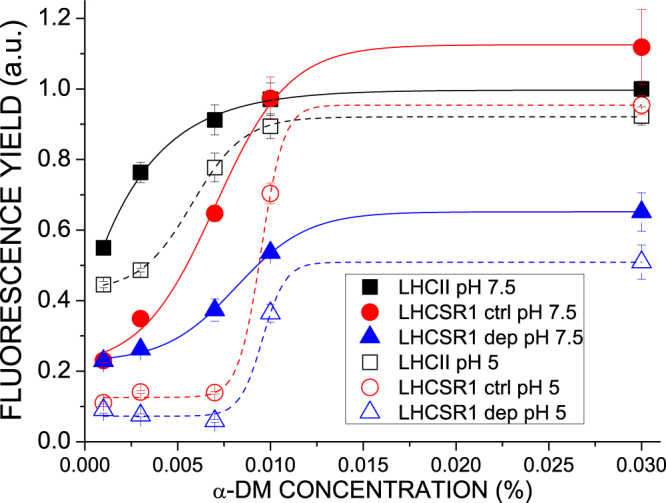
Fluorescence quantum yield at different pH and detergent concentrations of LHCII as compared to  LHCSR1 control and de-epoxidized. Distributions of fluorescence quantum yields at the different detergent concentrations were fitted with sigmoidal functions.

Fluorescence quantum yield of LHCSR1-ctrl/dep was analyzed and compared to that of trimeric LHCII. Since the interaction with detergents negatively influences the transition to a dissipative state of LHC proteins by preventing protein-protein interactions^13, 16, 55–59^ measurements were performed at different α-DM concentrations, ranging from 0.03% to 0.001%. Also, the protein solution was buffered at either pH 7.5 or pH 5.0 to mimic triggering of quenching *in vivo* by thylakoid lumen acidification. As reported in Fig. 3, LHCSR1 exhibited a sigmoidal transition from a highly fluorescent state to a quenched state with decreasing detergent concentration. Fluorescence yield at pH 7.5 and high detergent was similar for LHCII and LHCSR1-ctrl, while it was 50% lower for LHCSR1-dep. A moderate quenching was induced by acidification which was weak for LHCII (5%) and stronger for LHCSR1, either -ctrl or -dep. Decreasing detergent concentration on LHCII caused further quenching to a final yield of 50% compared to the yield at 0.03% DM. The effect on LHCSR was stronger with both -ctrl and -dep samples retaining a 20% fluorescence yield at 0,003% DM with respect to LHCSR ctrl at 0,03% DM. Decreasing detergent at pH 5.0 led to a further decrease in fluorescence and the transition from high to low fluorescence states was sharper, irrespective of the de-epoxidation state of LHCSR. This effect was also observed with LHCII and yet the transition was smoother and the shift to low fluorescent state was not quite complete even at 0,003% DM in the latter. LHCSR1-dep exhibited a lower fluorescence yield compared to LHCSR1-ctrl at all conditions tested, with the exception of the most extreme quenching conditions at 0.001% α-DM, where the differences were lost. By applying sigmoidal fitting of the detergent-dependent transition from high to low fluorescent state of the different complexes, the *x*_0_ values, i.e. detergent concentrations at which half of the subunits are found in quenched state (Table 3), were extrapolated. *x*_0_ values were always higher in LHCSR1 proteins compared to LHCII, with low pH further increasing the quenched fraction (Table 3). Zea binding increased *x*_0_ value at pH 7.5 but not at pH 5.0. These results indicate that protein-protein interactions, induced by detergent shortage, caused LHCSR1 to undergo transition to a dissipative state **﻿﻿far﻿﻿** more efficiently compared to LHCII: acidification activated an additional quenching effect in LHCSR1-ctrl and -dep, while a weaker response to low pH was evidenced in LHCII.

**Table 3:** *x*_*0*_ values of sigmoidal fitting of fluorescence quantum yield dependency on detergent concentrations.

	x_0_	s.d.
LHCII 7.5	−0.0223	*0*.*0170*
LHCII 5.0	0.0058	*0*.*0001*
LHCSR1 ctrl 7.5	0.0070	*0*.*0006*
LHCSR1 ctrl 5.0	0.0095	*0*.*0002*
LHCSR1 dep pH 7.5	0.0082	*0*.*0003*
LHCSR1 dep pH 5.0	0.0096	*0*.*0001*

***x***_*0*_ parameter obtained upon sigmoidal fitting of curves reported in Fig. 3 is indicated with standard deviation (s.d.).

### Fluorescence lifetime analysis

**Figure 4:**
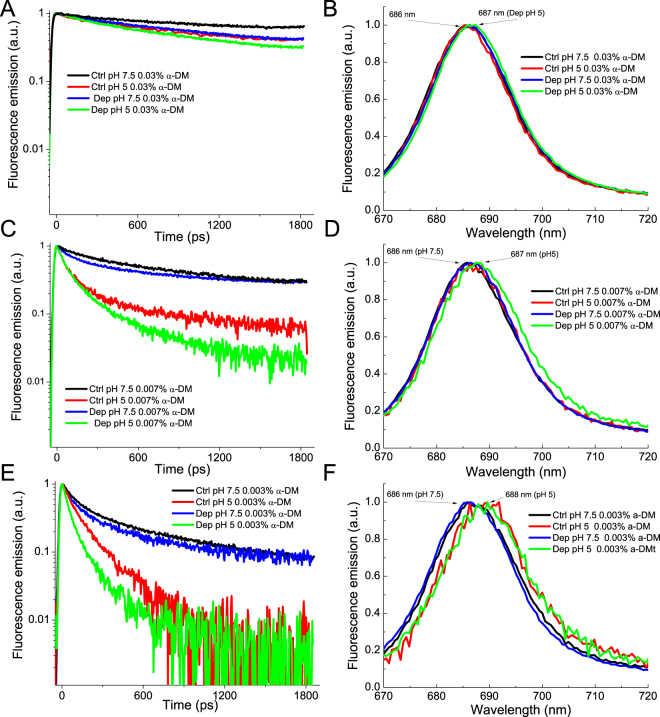
Fluorescence decay kinetics and integrated spectra of LHCSR1 CTRL and DEP at different pH and detergent concentration. Fluorescence decay kinetics were measured for LHCSR1 CTRL and DEP at three detergent concentrations (0.03%, 0.007% and 0.003% α-DM, Panels (A), (C) and (E) respectively) at two different pH (7.5 and 5). Integrated spectra are reported in Panel (B), (D) and (F).

In order to gain information on the quenched states induced in LHCSR1 proteins, florescence lifetime measurements were performed on samples at selected α-DM concentrations and pH-values using a time resolved spectroscopy system based on Streak camera detection. Visual inspection of the fluorescence decay kinetics integrated on 670–800 nm (Fig. 4) showed faster dynamics with decreasing detergent concentration. At any detergent concentration, decay curves were faster in presence of Zea and faster decay components were observed at pH 5 compared to pH 7.5 for both -ctrl and -dep samples. The fluorescence emission spectra obtained by integrating the emission over 0–1800 ps time range were very similar and yet small changes could be detected: in the case of LHCSR-dep pH 5 at 0.007% α-DM showed a broadening by 2 nm of the FWHM, specifically towards lower energies. This pH dependent red-shift of fluorescence emission was stronger at 0.003% α-DM and extended to LHCSR1-ctrl sample. In the case of the LHCII fluorescence decay curves a pH response was not evident at 0.03% detergent concentration, but only upon partial protein aggregation at 0.007% and 0.003% α-DM concentration (Supplementary Figure S1). While at 0.03% α-DM LHCSR1 and LHCII decay curves were similar (Supplementary Figure S1 and Fig. 4), at lower detergent concentration LHCII fluorescence lifetimes were always longer compared to LHCSR1 samples, in agreement with fluorescence quantum yield results reported in Fig. 3. The integrated fluorescence emission spectra obtained for LHCII samples did not show any significant dependence on pH or detergent concentration (Supplementary Figure S1).

**Figure 5:**
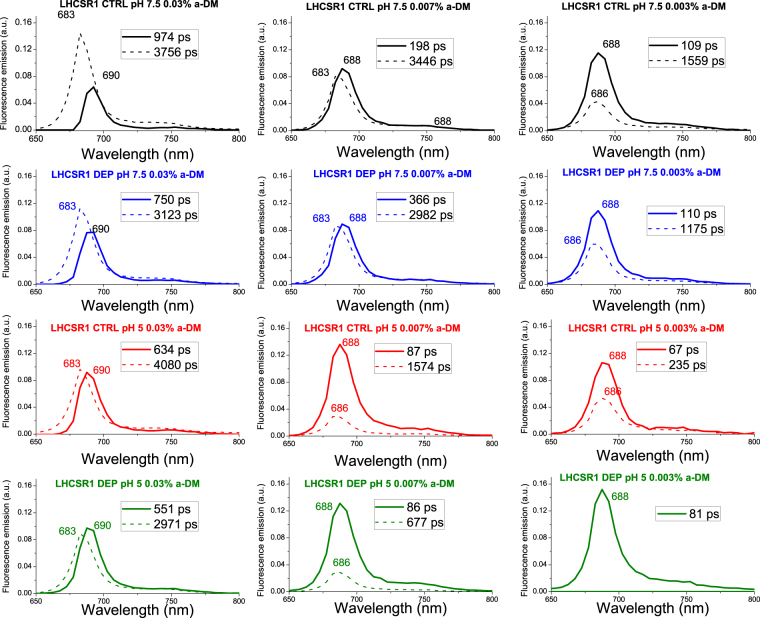
Decay Associated Spectra (DAS) resulting from global analysis of LHCSR1 fluorescence decay maps. Streak camera fluorescence decay maps recorded for LHCSR1 CTRL and DEP at different detergent concentration (0.03%, 0.007% and 0.003% α-DM) and pH (7.5 and 5) were fitted with a global analysis method with two exponential functions. DAS obtained are reported, in dashed line the longest component and in solid line the shortest.

Fluorescence decay maps were then fitted by global analysis as described in ref. 60 using a bi-exponential decay function with the only exception of LHCSR1-dep measured at pH 5.0 and 0.003% DM, where one exponential function was sufficient for best fitting. Employing three exponentials did not significantly improve the quality of the fit. The wavelength-dependent amplitudes of the decay components were then used to reconstruct the decay associated spectra (DAS) which are reported in Fig. 5.

When analyzing the DAS of LHCSR1-ctrl and -dep samples, two spectral components could be resolved, with the shorter living form red-shifted (688–690 nm) compared to the long living one (683–686 nm). Lifetime of both forms shortened with decreasing detergent concentration: τ1 decreased from 974 ps to 67–80 ps, with the shortest lifetime value found at pH 5.0 both in LHCSR1-ctrl and -dep samples. As for the amplitudes, those associated to the long living components decreased, while the weight of short living forms increased with lowering the detergent concentration and/or the pH. The τ2 lifetime ranged from 4 ns at pH 7.5 to sub-ns (0.24–0.67 ns) at low detergent concentration and pH 5.0. Zea binding at any condition analyzed caused a significant reduction of the amplitudes of the DAS with the longer time constants. The shorter time constants were always characterized by higher amplitude in LHCSR1-dep compared to LHCSR1-ctrl, with the 80 ps component peaking at 688 nm being the only DAS detected from the LHCSR1-dep at 0.003% and pH 5 (Table 4). This is consistent with the presence of two distinct conformations of LHCSR that can be interconverted into each other depending on detergent concentration with Zea favoring the fast decaying conformation and enhancing the pH response. An example of the Zea-dependent reduction of the long-living component can be appreciated when comparing LHCSR-ctrl vs LHCSR-dep at 0.007% α-DM (Table 1), whose lifetime is further reduced at low pH, to a minimum of 677 ps in LHCSR1-dep pH 5.0. Transition of LHCSR1 to a dissipative state is thus correlated with a red-shift of fluorescence emission and formation of quenched states with associated fluorescence decay time constants below 100 ps. In the case of LHCII, fluorescence decay maps were also globally fitted with two components, whose amplitudes and time constants are reported in Supplementary Table S1, while the DAS are shown in Supplementary Figure S2. Again, the amplitude and the time constant of the longer components (3.9–1.4 ns) decreased with reducing the detergent concentration while the amplitude of the short lifetime component (241–407 ps) increased, even more at pH 5.0 in α-DM 0.007% and 0.003%. The two DAS obtained from LHCII decays were spectrally very similar, peaking at 683 nm. This was different compared to LHCSR1, and consistently no very fast components (namely < 100 ps) were resolved in LHCII (Supplementary Table S1, Supplementary Figure S2). Finally, the shortest average lifetimes of LHCII was 690 ps at 0.003% α-DM and pH 5.0, compared to the 127 and 81 ps values found in the case of LHCR1-ctrl and LHCSR1-dep respectively, in agreement with a special role of LHCSR1 as a quencher of excitation energy.

**Table 4:** Results of global analysis of fluorescence decay kinetics on isolated proteins.

Sample	A1	τ1 (ps)	A2	Τ2 (ps)	τavg (ps)
LHCSR1 CTRL pH 7.5 0.03% a-DM	26%	974	74%	3756	3042
LHCSR1 DEP pH 7.5 0.03% a-DM	39%	750	61%	3123	2198
LHCSR1 CTRL pH 5 0.03% a-DM	44%	634	56%	4080	2567
LHCSR1 DEP pH 5 0.03% a-DM	52%	551	48%	2971	1720
LHCSR1 CTRL pH 7.5 0.007% a-DM	52%	198	48%	3446	1742
LHCSR1 DEP pH 7.5 0.007% a-DM	49%	366	51%	2982	1687
LHCSR1 CTRL pH 5 0.007% a-DM	81%	87	19%	1574	372
LHCSR1 DEP pH 5 0.007% a-DM	82%	86	18%	677	192
LHCSR1 CTRL pH 7.5 0.003% a-DM	69%	109	31%	1559	553
LHCSR1 DEP pH 7.5 0.003% a-DM	64%	110	36%	1175	495
LHCSR1 CTRL pH 5 0.003% a-DM	64%	67	36%	235	127
LHCSR1 DEP pH 5 0.003% a-DM	100%	81	—	—	81

2D Streak camera maps were fitted with a bi-exponential decay function with a global analysis method. Amplitude and time constants are reported. Average fluorescence lifetimes were calculated as ΣA_i_τ_i_/ΣA_i_.

**Figure 6:**
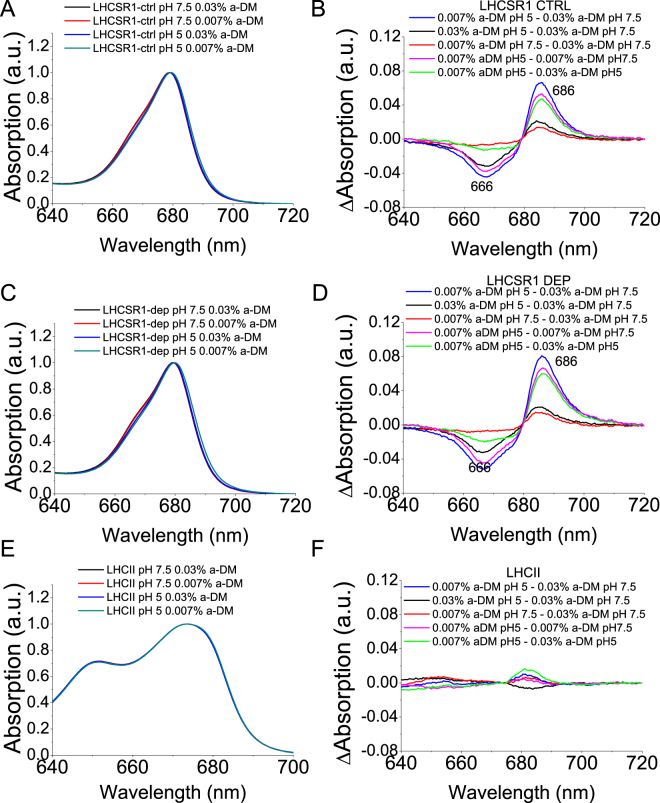
Absorption spectra of LHCSR1 and LHCII at different detergent concentration and pH. Panels A,C,E: LHCSR1 CL (Panel A), LHCSR1 DEP (Panel C) and LHCII (Panel E) at pH 7.5 or pH 5 at α-DM 0.03% or 0.007% concentration. Panel B,D,E: difference spectra for LHCSR1 CL (Panel B), LHCSR1 DEP (Panel D) and LHCII (Panel F).

**Figure 7:**
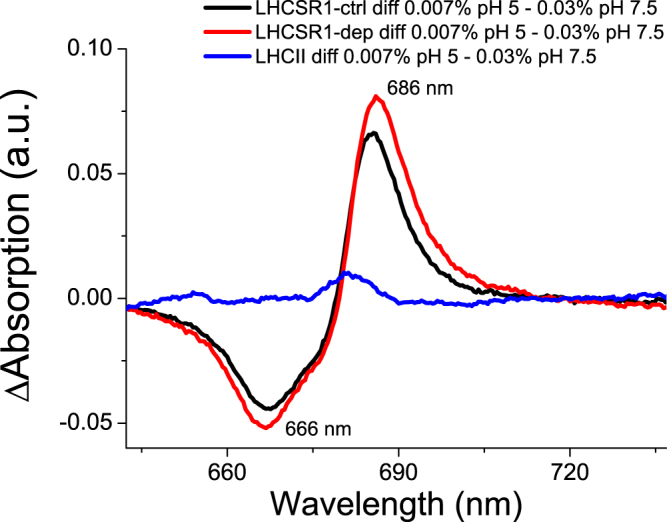
Difference absorption spectra of LHCSR1 and LHCII between the condition of low detergent and low pH and the condition of high detergent and high pH.

To further characterize the formation of red-shifted quenched states, we recorded the absorption spectra of LHCSR1-ctrl and LHCSR1-dep at both pH 7.5 and at pH 5 and at 0.03% vs 0.007% α-DM and compared them to those of LHCII measured in the same conditions. The Qy transition of LHCSR1-ctrl and LHCSR1-dep was red-shifted at pH5.0, 0.007% α-DM compared to other conditions, while no shift was observed in the case of LHCII (Fig. 6). The difference spectra reported in Fig. 6B and D were calculated by subtracting in each case the absorption spectrum of a less quenched sample from that of a more quenched sample. These were all characterized by negative/positive signals with negative terms at 667 nm and positive ones at 686 nm, likely due to a red-shift of the absorption, possibly connected to the formation of the quenched state. The effect of detergent reduction at pH 5 (green lines in Fig. 6B and C) was similar to the effect of pH reduction at 0.007 α-DM (pink lines in Fig. 6B and C), with the stronger differences associated to the combination of acidification and low detergent concentration (blue lines in Fig. 6B and C). Reducing detergent concentration at pH 7.5 or, conversely, reducing pH at 0.03% α-DM, instead, yielded a far weaker effect on absorption properties of LHCSR1 proteins (red and black lines, respectively). LHCII did not respond to the same changes in pH and/or detergent as judged from the small amplitude of the difference spectra (Fig. 6F). Figure 7 reports the difference spectra obtained subtracting the less quenched state (0.03% α-DM at pH 7.5) from the more quenched state (0.007% α-DM at pH 5.0) for LHCSR1 and LHCII, showing significant changes in the case of LHCSR1 only. Also, Zea binding to LHCSR1 caused a difference spectrum similar to that observed with LHCSR1-ctrl and yet amplitude was enhanced. Similar results were obtained upon deconvolution of LHCSR1-ctrl and LHCSR1-dep absorption spectra with Chl spectral forms in the Qy region: Chl a spectral forms peaking at 683.9 and 688.9 nm were increased at pH 5 at 0.007% α-DM, further increased in presence of Zea (Supplementary Figure S3).

## Discussion

In this work we have investigated the spectroscopic properties and quenching activity of isolated LHCSR1 protein either in its Vio or Zea-binding form. Zea accumulation in *P*. *patens* was previously shown to cause a tenfold enhancement of LHCSR1 activity *in vivo* ^33^. Deconvolution of absorption spectra in the Soret region (Fig. 2) showed multiple absorption forms for carotenoid spectral components (Table 2, Fig. 2) which were interpreted based on a chromophore complement of 8 chlorophyll a and 4 xanthophylls^49^. This implies that the two xanthophyll species detected by HPLC analysis (Table 1), Lute and Vio, each bind to more than one site within LHCSR1. Each site within LHC proteins is characterized by a different refractive index causing a specific shift with respect to the absorption in organic solvent. The L2 site induces the strongest red shift^45, 54, 61^. Indeed, absorption forms accounting for half of the total absorption were strongly red-shifted and could be further divided in two groups with shift of, respectively, 17.4 and 20 nm and similar amplitude (Table 2), consistent with location in site L1 and L2, respectively. Interestingly, the L1 site was previously shown to have single occupancy, hosting Lute in both LHCII and CP29^5, 6^. Instead, L1 site of LHCSR1 appeared to bind both Lute and Vio (87%/13%) as judged by the extraction of a significant fraction of Vio absorption with a 12.6 nm shift. Site L2 was almost fully occupied by Vio, similar to the case of CP29^6^. The remaining 50% of carotenoid absorption was characterized by far lower red-shift compared to its absorption in organic solvent (9–10 nm) consistent with a more peripheral location in the protein structure. Peripheral sites in LHCb proteins were identified with sites V1 and N1^5, 45, 62^ and could be distinguished in deconvolutions due to the distinct absorption of Neoxanthin (Neo) and Vio. Lack of Neo in LHCSR1 makes discrimination between occupancy of sites L1 and N1 difficult and yet absorption form of both Vio and Lute were resolved with weakly red-shifted absorptions. Upon de-epoxidation, two different Zea forms were resolved: a 15.5 nm red-shifted form, which is thus attributed to the inner L2 site, and a 6 nm shifted form attributed to a peripheral carotenoid binding site, either a V1-like or N1-like site. Interestingly, de-epoxidation completely suppressed the 8 nm shifted Vio forms, suggesting that Vio in peripheral site was completely substituted with Zea. Also, no 10–12 nm shifted Zea forms were detected, implying Zea did not bind to site L1 as previously reported in the case of LHCII^61^. Replacement of Vio by Zea upon de-epoxidation has been previously reported to occur more rapidly and completely in peripheral site V1 as compared to the inner L2 or L1 sites^11, 43^ consistent with the present finding of a 50% ligand replacement by Zea in site L2. It should, however, be considered that *P*. *patens* undergoes faster and more complete de-epoxidation with respect to tobacco^33^ suggesting that occupancy by Zea of the L2 site could well be higher than 50% or complete in high light-exposed mosses. Zea has been previously reported both to act as an allosteric modulator favoring transition of LHC proteins to a dissipative state and/or to directly participate to quenching reactions. In both cases site L2 was the suggested binding site^37, 38, 56, 63^. To support this view, LHCII trimers with V1 site occupied by Vio *vs* Zea were found to exhibit the same fluorescence yield^45, 47, 64^. This was contrasting with findings on LHCSR1: Zea vs Vio binding to LHCSR1 caused a ~50% reduction in Chl fluorescence yield at high detergent at either neutral or acidic pH. The Zea-dependent quenching effect was maintained with decreasing detergent concentration, finally reaching the same yield of quenched state only at 0,001% DM. Zea binding to LHCSR1 led to transition to a dissipative state^29^ which was enhanced at low pH. Interestingly, the dissipative state was associated to the appearance of low energy chlorophyll absorption and fluorescence emission forms in both LHCSR1-ctrl and LHCSR1-dep, which is strengthened by Zea (Figs 3 and 6). In the case of LHCII, no low energy absorption or emission form was detected by decreasing detergent concentration or pH. Low-energy excited states have been attributed to increased chlorophyll-chlorophyll excitonic interactions, which suggests the possible formation in LHCSR1 of a quenching site by increasing the strength of Chl-Chl interactions, as previously reported for CP29^38^. Zea-dependent quenching in LHCSR1 has been recently attributed to energy transfer from chlorophyll(s) to the short-living S1 state of carotenoids^29^. However, the fluorescence lifetime of LHCSR1-dep at pH 5.0 at 0.03% α-DM was 1.7 ns, with the shortest component decaying with a time constant of 551 ps. Such near-ns time ranges are unsuitable for physiological quenching activity: indeed, LHCSR1 was reported to be an effective quencher for both PSI and PSII, which have lifetimes of 50–60 and 100–200 ps respectively^2, 3, 65^. A pH dependent quenching effect was also reported for *C*.*r*.LHCSR3 in 0.03% α-DM or in nanopolymers^17^ with time constants in the ns time range, unsuitable for explaining quenching activity *in vivo*. Shorter lifetimes were obtained by reducing the detergent concentration of LHCSR1, either in presence or absence of Zea. This effect was also observed with LHCII (Fig. 3, Supplementary Figure S1) in agreement with a previous report^55^ but transition to low fluorescence state was far less complete and required a stronger detergent depletion with respect to the case of LHCSR1, as evident from the higher *x*_0_ value, (Table 3). LHCSR1 fluorescence lifetime could become as short as 81 ps in presence of Zea at low detergent and pH 5.0, while in the case of LHCII lifetimes longer than 600 ps were measured, with the shorter components living longer than 240 ps. Transition to dissipative state was facilitated and more evident at pH 5.0, allowing for LHCSR1 to reach a strongly quenched conformation that could not be reached at pH 7.5. Zea binding to LHCSR1 caused a stronger quenching at each detergent concentration compared to LHCSR1-ctrl, with the exception of 0.001% α-DM, where both the LHCSR1-ctrl and LHCSR1-dep were quenched to a similar strong level. Low pH and protein-protein interactions, induced by lowering detergent concentration, are thus the driving force leading LHCSR1 to a dissipative state, while Zea tuned the equilibrium between unquenched and quenched states toward the latter. The observation that *in vivo* Zea accumulation is required for NPQ activation in *P*. *patens* while a strong quenching can be observed *in vitro* even in absence of Zea, could be due to the different conditions experienced by LHCSR1 in thylakoids compared to *in vitro*. Proteins aggregation *in vitro* was indeed suggested to mimic the protein-protein interactions experienced by LHC proteins *in vivo*. Nevertheless, interactions *in vivo* are established with specific partners^66, 67^ providing complementary domains which might well modulate aggregation conditions differently respect to the case of the LHCSR1-only system induced *in vitro*. *In vivo*, Zea could be required for a complete transition to a dissipative state, as observed *in vitro* at intermediate detergent concentration. We cannot indeed exclude that LHCSR1 activity *in vivo* requires specific protein-protein interactions that in *vivo* are established in presence of Zea only alike *in vitro* at intermediate detergent concentration, while at low pH and low detergent concentration the strong protein aggregation might mimic the effect of Zea. A further hypothesis is that *in vivo* the activity of LHCSR1 as a quencher might depends on Zea binding due to a specific Zea-dependent quenching mechanism as recently observed for the LHCII-dependent NPQ in Arabidopsis^68^. *In vitro*, both Zea-dependent and Zea-independent quenching can be observed (Figs 3 and 4), with the former being more efficient unless the most extreme conditions (pH 5, 0.001% α-DM) where the quenching is similar. Correlation between *in vitro* and *in vivo* conditions can be obtained based of lifetime of the most abundant thylakoid component, i.e the major LHCII, which has been determined to be ~2 ns^69^ in thylakoid membranes. Lifetime in this range for LHCII is observed at detergent concentrations between 0,007% and 0,010% (Supplementary Table S1) suggesting the conditions experienced by LHCSR1 *in vivo* corresponds to *in vitro* conditions where the Zea-dependence of quenching is strong; suggesting that Zea could be critical for transition from conservative to dissipative conformations at lumenal pH values intermediate between 5 and 7.5 (Figs 3 and 4).

**Figure 8:**
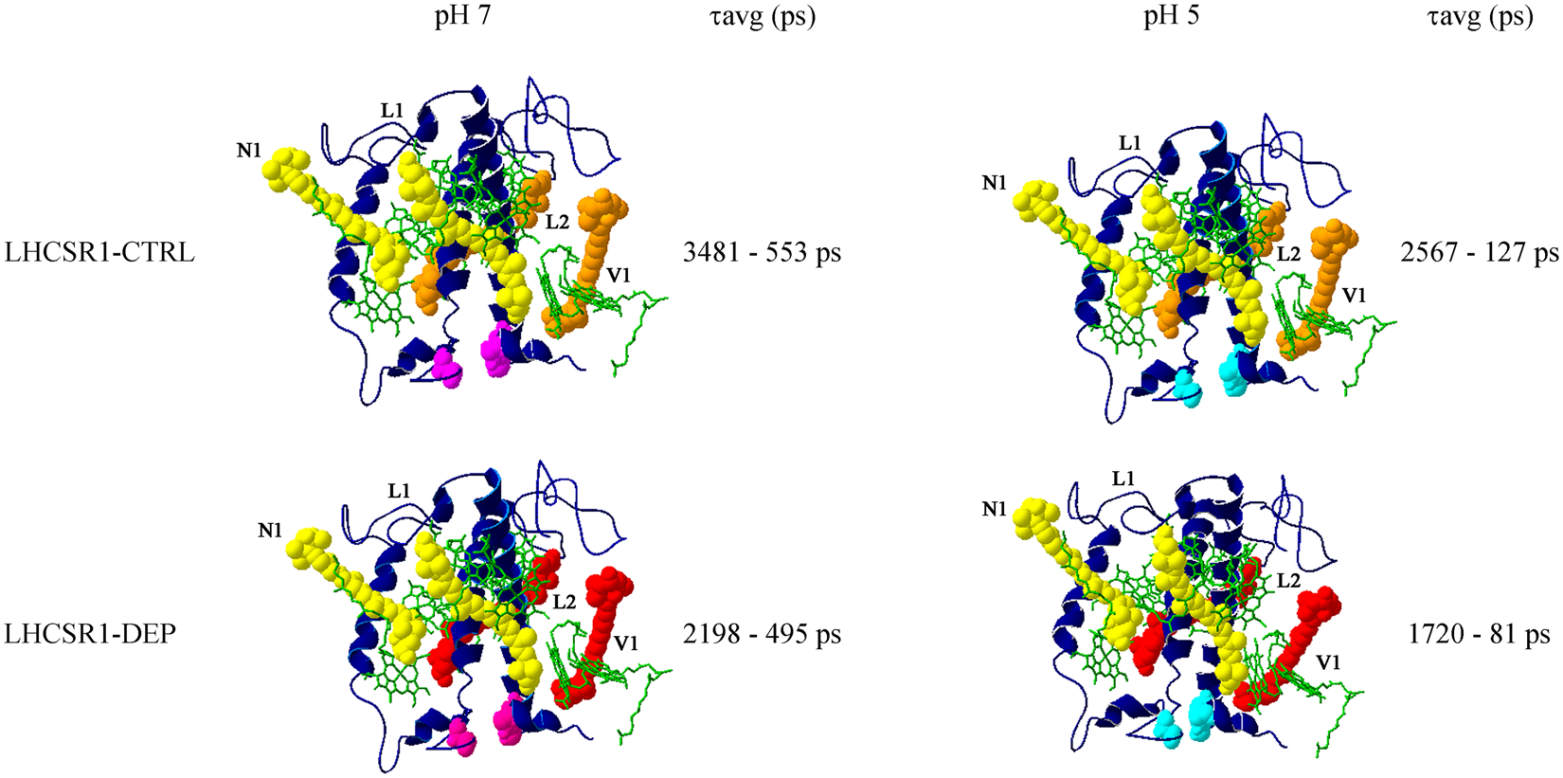
Proposed model for LHCSR1 conformational change during activation of quenching. LHCSR1 structure was modeled based on crystallographic structures of LHCII and CP29. The average fluorescence lifetimes (τ_AVG_) at low and high detergent concentration and a different pH are shown for both LHCSR1-ctrl (upper panel) and -dep (lowe panel); green, chlorophylls; yellow, lutein; orange, violaxanthin; red, zeaxanthin. Prononatable residues exposed to the lumenal side are indicated in pink and at pH 7.5 and in cyan at pH 5.0. The 3D structure is obtained by homology with CP29 and LHCII and does not account for the structural changes undergone during quenching activation: it is only meant to depict the sites of xanthophyll conversion.

In conclusion, this work shows that Zea, produced upon excess illumination by the luminal enzyme VDE from pre-existing Vio in conditions of low pH, binds to both peripheral and inner sites of the photoprotective protein LHCSR1 (Fig. 8). At detergent conditions which favor protein-protein interactions, low pH causes conformational change(s) through protonation of lumenal-exposed residues^17, 20^ shifting LHCSR1 to a quenched state. Acidification appears as the main trigger in this process since it is effective with both dep- and ctrl- LHCSR1. Zea enhances the efficiency of LHCSR1 switching to its dissipative state, characterized by formation of low energy chlorophyll absorption forms. LHCSR1 in a dissipative state is red-shifted and has a short lifetime (~80 ps in the case of Zea binding samples) allowing for LHCSR1 to compete with the PSII reaction center for excitation energy localized in the LHC antenna system. In the case of PSI, LHCSR1 quenching state is hardly competitive with the fast charge separation occurring in PSI reaction centers^2^. However, the 77 K fluorescence analysis has shown that activation of quenching by LHCSR1 decreases the amplitude of PSI-LHCI emission in *P*. *patens*, specifically quenching a portion of the bound antenna proteins^65^. This suggests LHCSR1 does not quench PSI-LHCI directly but rather modulates the lifetime of a LHCII population resident in stroma membranes which enhances PSI antenna size in non-quenching conditions. It is yet unclear whether LHCSR1 directly competes with PSI when in its quenching state within a functional PSI-LHCI-LHCII-LHCSR1 supercomplex or whether LHCSR1 competes with PSI–LHCI for binding to LHCII complexes. The present determination of ~80 ps as the shortest average fluorescence lifetime of LHCSR1 in its quenched state supports the latter hypothesis.

## Methods

### Sample preparation and pigment analysis

The P.p.LHCSR1 was obtained from *Nicotiana tabacum* plants transformed with the LHCSR1 gene sequence from *P*. *patens* as previously reported^49^. Zea binding LHCSR1 protein was isolated upon *in vitro* de-epoxidation of thyalkoids extracted from LHCSR1 overexpressing *Nicotiana tabacum* plants^49^. LHCII trimers were purified from tobacco as described in ref. 45. Pigments were extracted in acetone 80% and analyzed by deconvolution of absorption spectra with carotenoid and chlorophyll spectral forms in acetone 80% to determine Chl a/b ratio and Chl/Car ratio. HPLC pigment analysis was used as a constraint in spectral deconvolution as previously reported in ref. 50.

### Absorption and Fluorescence spectra

Absorption spectra were measured by DW2000 Aminco spectrophotometer and deconvoluted as described in refs 54 and 70. In particular absorption spectra in the 600–750 nm were fitted using Chl a and Chl b spectral forms in protein environment as described in ref. 70. In the case of the Soret region,the absorption spectra in the 400–520 nm range were fitted with Chl and Car spectral forms properly shifted compared to absorption acetone 80% as described in ref. 54. The number of Chl and Car spectral forms and the shift associated to the reference spectral forms were determined by the fitting procedure. The quality of the fit was evaluated by Ordinary Least Squares analysis and considering the correspondence of Chl a/b ratios obtained by deconvolution of native spectra and by pigment analysis. 77 K steady state emission spectra were measured as reported in ref. 71.

### Time resolved fluorescence measurements

Time-resolved fluorescence measurements were performed using a femtosecond laser excitation source and a streak camera detection system, as reported in ref. 36.

### Global analysis

Streak camera 2D fluorescence decay maps were globally fit with exponential functions as previously reported^36, 60^.

### Data availability

All the data generated during and/or analysed during the current study are available from the corresponding author on reasonable request.

## Electronic supplementary material

Table S1, Figure S1, Figure S2
