# Supplementary material for: Functional modulation of LHCSR1 protein from Physcomitrella patens by zeaxanthin binding and low pH
Source: Sci Rep. 2017 Sep 11;7:11158. doi: 10.1038/s41598-017-11101-7 (PMC5593824; doi:10.1038/s41598-017-11101-7)
Supplement: Supplementary file 1 — Table S1, Figure S1, Figure S2 [file 41598_2017_11101_MOESM1_ESM.pdf]

Functional modulation of LHCSR1 protein from *Physcomitrella patens* by zeaxanthin binding and low pH

Alberta Pinnola[1], Matteo Ballottari[1], Ilaria Bargigia[2], Marcelo Alcocer[2], Cosimo D'Andrea[2,3], Giulio Cerullo[3] and Roberto Bassi[1,4*]

[1] Dipartimento di Biotecnologie, Università di Verona, Strada Le Grazie 15, I-37134 Verona, Italy

[2] Center for Nano Science and Technology @PoliMi, Istituto Italiano di Tecnologia, via Pascoli 70/3, 20133 Milano, Italy

[3] IFN-CNR, Department of Physics, Politecnico di Milano, P.za L. da Vinci 32, 20133 Milano, Italy

[4] Consiglio Nazionale delle Ricerche (CNR), Istituto per la Protezione delle Piante (IPP), Via Madonna del Piano 10, 50019 Sesto Fiorentino, Firenze, Italy.

[*]Corresponding author: roberto.bassi@univr.it

SUPPLEMENTAL DATA

Table S1: Results of global analysis of fluorescence decay kinetics on LHCII isolated proteins. 2D Streak camera maps were fitted with two exponential function with a global analysis method. Amplitude and time constants are reported. Average fluorescence lifetimes were calculated as $\Sigma A_i \tau_i / \Sigma A_i$

**SAMPLE**	**A1**	**τ1 (ps)**	**A2**	**τ1 (ps)**	**τavg (ps)**
**LHCII pH 7.5 0.03% a-DM**	16%	241	84%	3753	3195
**LHCII pH 5 0.03% a-DM**	19%	297	81%	3933	3249
**LHCII pH 7.5 0.007% a-DM**	24%	304	76%	3871	2992
**LHCII pH 5 0.007% a-DM**	58%	407	42%	3403	1654
**LHCII pH 7.5 0.003% a-DM**	53%	325	47%	3069	1604
**LHCII pH 5 0.003% a-DM**	76%	395	24%	1373	630

Figure S1: Fluorescence decay kinetics and integrated spectra of LHCII at different pH and detergent concentration. Fluorescence decay kinetics were measured at three detergent concentrations (0.03%, 0.007% and 0.003% α-DM, Panels A, C and E respectively) at two different pH (7.5 and 5). Integrated spectra are reported in Panel B, D and E.

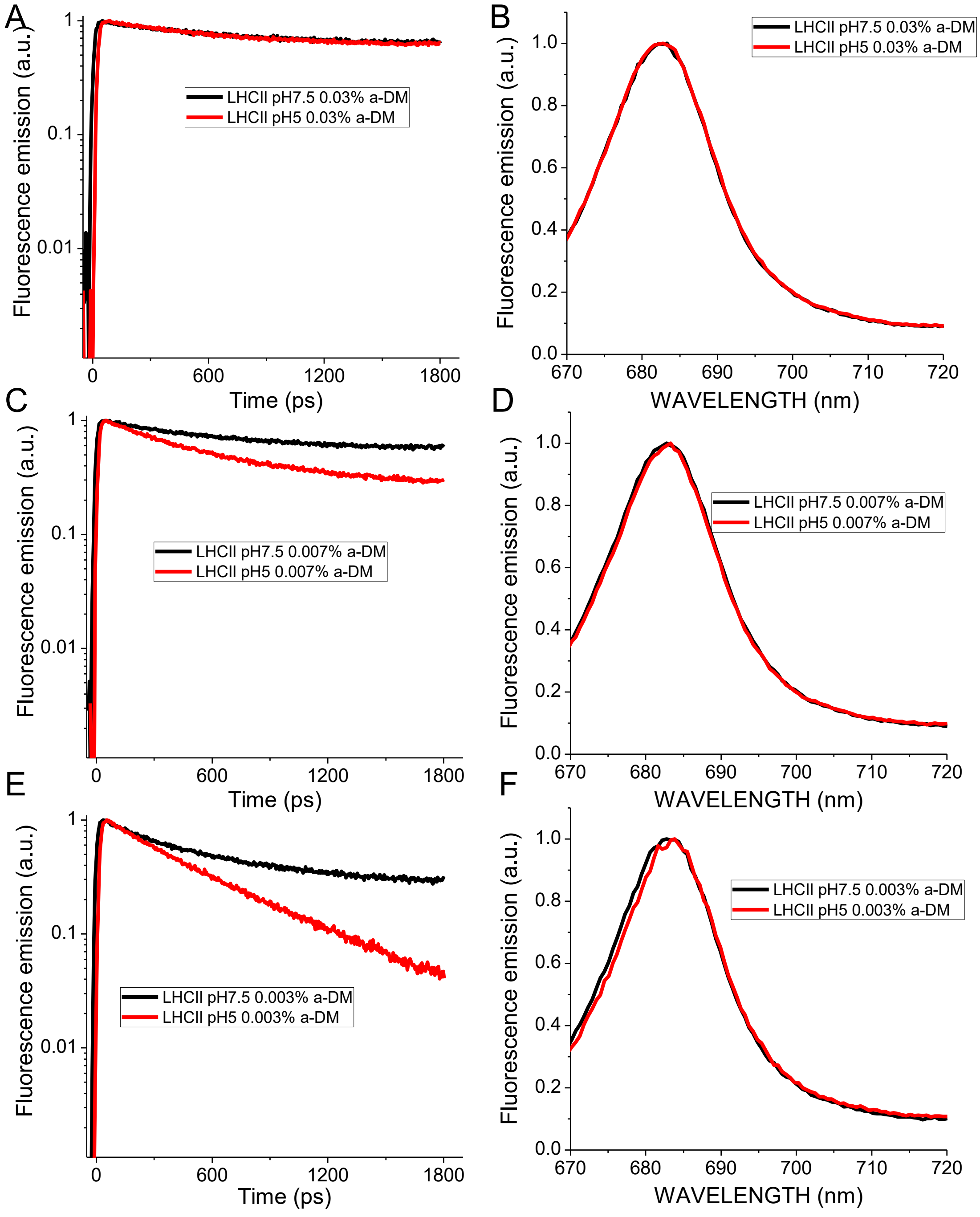

Figure S2: Decay Associated Spectra (DAS) resulting from global analysis of LHCII fluorescence decay maps. Streak camera fluorescence decay maps recorded for LHCII at different detergent concentration (0.03%, 0.007% and 0.003% α-DM) and pH (7.5 and 5) were fitted with a global analysis method with two exponential functions. DAS obtained are reported, in dashed line the longest component and in solid line the shortest.

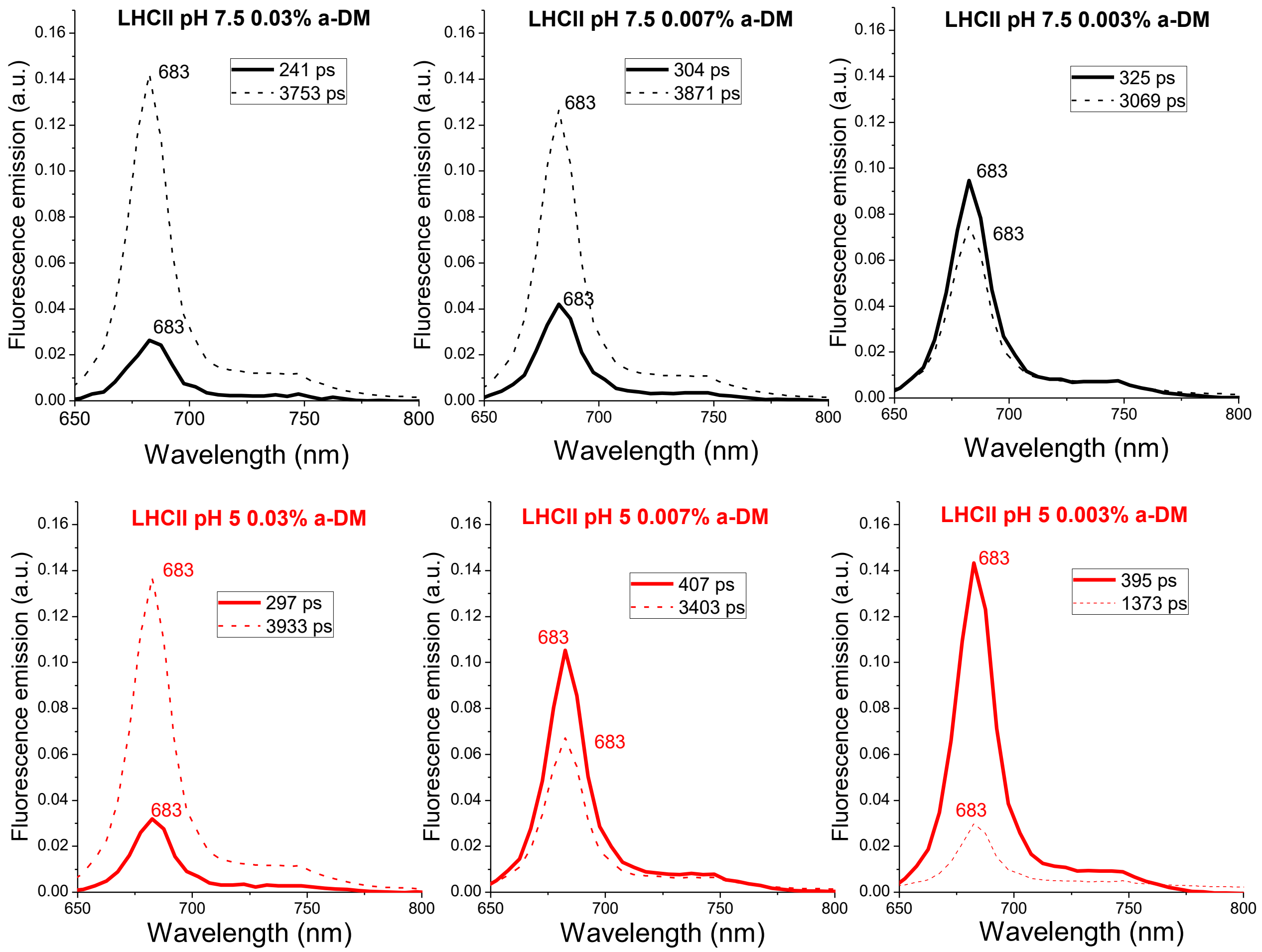

Figure S3: Deconvolution of LHCSR1 absorption spectra in Qy region. LHCSR1-ctrl and -dep absorption spectra in the red region at pH 7.5 or pH 5 at 0.003% or 0.007% were fitted with three chlorophyll b and seven chlorophyll a spectral forms. The amplitude of the chlorophylls spectral forms are reported in Panel I and J for LHCSR1-ctrl and -dep respectively.

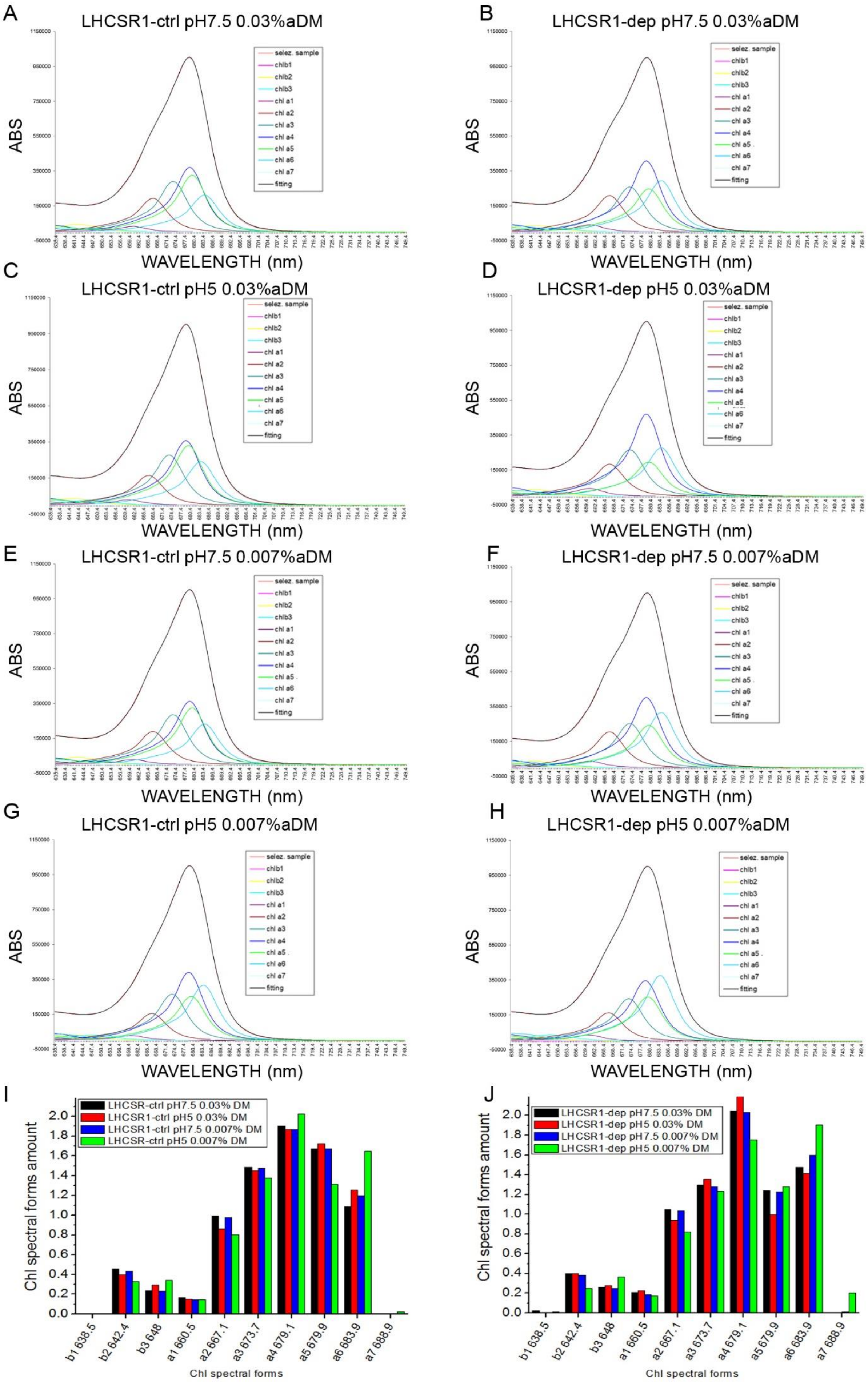
A
LHCSR1-ctrl pH7.5 0.03%aDM
B
LHCSR1-dep pH7.5 0.03%aDM
C
LHCSR1-ctrl pH5 0.03%aDM
D
LHCSR1-dep pH5 0.03%aDM
E
LHCSR1-ctrl pH7.5 0.007%aDM
F
LHCSR1-dep pH7.5 0.007%aDM
G
LHCSR1-ctrl pH5 0.007%aDM
H
LHCSR1-dep pH5 0.007%aDM
ABS
WAVELENGTH (nm)
selez. sample
chlb1
chlb2
chlb3
chl a1
chl a2
chl a3
chl a4
chl a5
chl a6
chl a7
fitting
I
LHCSR-ctrl pH7.5 0.03% DM
LHCSR1-ctrl pH5 0.03% DM
LHCSR1-ctrl pH7.5 0.007% DM
LHCSR-ctrl pH5 0.007% DM
J
LHCSR1-dep pH7.5 0.03% DM
LHCSR1-dep pH5 0.03% DM
LHCSR1-dep pH7.5 0.007% DM
LHCSR1-dep pH5 0.007% DM
Chl spectral forms amount
Chl spectral forms
b1 638.5
b2 642.4
b3 648
a1 660.5
a2 667.1
a3 673.7
a4 679.1
a5 679.9
a6 683.9
a7 688.9
